# Atypical Neuromyelitis Optica Spectrum Disorder: A Case of Unilateral Optic Neuritis and Partial Transverse Myelitis

**DOI:** 10.7759/cureus.95461

**Published:** 2025-10-26

**Authors:** Alaweya A Ofash, Muhannad Hashim, Ahmed Abbas, Omnia Ahmed, Osama S Abbadi, Faris Abdon

**Affiliations:** 1 Internal Medicine, Port Sudan Teaching Hospital, Port Sudan, SDN; 2 Department of Biochemistry, Faculty of Medicine, National University, Khartoum, SDN; 3 Department of Medical Biochemistry, Orotta College of Medicine and Health Sciences, Asmara, ERI

**Keywords:** aquaporin-4 antibodies, atypical presentation, autoimmune disorders, neuromyelitis optica, transverse myelitis, unilateral optic neuritis

## Abstract

Neuromyelitis optica spectrum disorder (NMOSD) is a rare autoimmune disease characterized by inflammation of the optic nerves and spinal cord, leading to optic neuritis and transverse myelitis. Atypical cases of NMOSD pose diagnostic challenges, particularly when initial symptoms deviate from classical presentations. We report an atypical NMOSD case involving a 22-year-old female presenting with unilateral optic neuritis and incomplete transverse myelitis. The patient exhibited myoclonus, acute urinary retention, and autonomic dysfunction. Diagnosis was confirmed through clinical assessment, ophthalmological evaluation, cerebrospinal fluid analysis, magnetic resonance imaging (MRI), and aquaporin-4 antibody testing. The patient responded positively to intravenous immunoglobulin and high-dose methylprednisolone, followed by maintenance azathioprine (rituximab planned as escalation if needed). This case underscores the crucial importance of early identification and timely serological testing in atypical presentations of NMOSD, particularly in resource-constrained settings.

## Introduction

Neuromyelitis optica spectrum disorder (NMOSD), first described by Devic in 1894 [1] and previously considered a severe variant of multiple sclerosis (MS), is now recognized as a distinct autoimmune astrocytopathy mediated by aquaporin-4 (AQP4) antibodies [2,3].

NMOSD primarily affects the optic nerves and spinal cord, causing significant disability without prompt intervention. Diagnostic criteria now allow definitive diagnosis with a single core clinical event combined with positive AQP4 serology [4,5].

Although classified as a rare disease, NMOSD shows striking geographic and ethnic heterogeneity. A 2021 systematic review of 33 population-based studies reported incidence and prevalence varying more than 20-fold worldwide, peaking in Afro-Caribbean and many Asian cohorts, and lowest in Australasia [6]. Women predominate by up to 9:1, and most patients present in early-to-mid adulthood [6,7]. Such data demand high clinical vigilance in non-white populations served by Middle Eastern and Northeast African centers.

Pathologically, AQP4-IgG activates complement and recruits granulocytes, leading to necrotizing astrocyte loss, secondary demyelination, and axonal injury [3,8]. Clinically, disability accumulates step-wise: untreated relapses leave more than half of patients blind or wheelchair-dependent within five years [3].

Atypical presentations, including unilateral optic neuritis and short-segment transverse myelitis, complicate timely diagnosis and treatment initiation. Importantly, the phenotypic spectrum continues to expand: unilateral optic neuritis, partial cervical myelitis, and isolated area-postrema syndrome can herald disease just as readily as the textbook pairing of bilateral optic neuritis with longitudinally extensive transverse myelitis (LETM) [8,9]. MRI red flags (posterior optic-chiasmal lesions, central LETM, bright-spotty cord lesions) and pronounced neutrophilic cerebrospinal-fluid pleocytosis help differentiate NMOSD from MS or other mimics [4,10].

Increased awareness of these atypical forms, particularly among high-risk ethnic populations, is crucial for optimal patient outcomes. This report contributes an atypical NMOSD case to the literature, emphasizing rapid identification and aggressive therapy in resource-limited healthcare environments.

## Case presentation

A 22-year-old Sudanese female presented with acute urinary retention, abdominal distension, and bilateral lower-extremity weakness. Three weeks earlier, she had transient blurred vision in the right eye that resolved spontaneously; no eye pain was documented. On general physical examination, she was afebrile and hemodynamically stable; cardiopulmonary and abdominal findings were unremarkable except for a palpably distended bladder.

On neurologic examination, mental status was normal (alert, oriented, normal speech and cognition). Cranial nerve testing showed a right internuclear ophthalmoplegia; pupils were equal and reactive. Funduscopy revealed mild temporal optic disc (nerve head) pallor on the right. Ishihara testing was not available; a bedside red-desaturation test showed reduced saturation in the right eye. Motor examination showed reduced tone in both lower limbs with Medical Research Council (MRC) grade 1/5 power in major lower-limb muscle groups bilaterally at nadir; upper-limb strength was 5/5. Sensory examination documented impairment in the lower limbs; a precise sensory level and individual modalities were not systematically recorded. Cerebellar testing (finger-to-nose) was intact in the upper limbs but limited in the lower limbs due to weakness. Deep tendon reflexes were reduced in the lower limbs; plantar responses and digital rectal examination were not documented. Autonomic involvement included urinary retention at presentation.

Serum studies showed values within reference ranges, including complete blood count (CBC), electrolytes, renal profile, and liver function tests (LFTs) (Table 1). Antinuclear antibody (ANA) and other autoimmune serology were not performed. Myelin oligodendrocyte glycoprotein (MOG)-IgG was not performed. Cerebrospinal fluid (CSF) analysis revealed normal glucose and protein levels; however, cell count/differential, IgG index, oligoclonal bands, and infectious studies (e.g., bacterial culture or viral polymerase chain reaction (PCR)) were not documented in the available medical record.

**Table 1:** Laboratory Findings with Corresponding Sudanese Reference Ranges

Laboratory Test	Patient Results	Reference Range
Hemoglobin	12.5 g/dL	12.0–15.5 g/dL
White Blood Cells (WBC)	6.2 × 10³/µL	4.0–11.0 × 10³/µL
Platelets	254 × 10³/µL	150–400 × 10³/µL
Serum Creatinine	0.8 mg/dL	0.6–1.2 mg/dL
Blood Urea Nitrogen (BUN)	13 mg/dL	7–20 mg/dL
Alanine Aminotransferase (ALT)	24 U/L	<35 U/L
Aspartate Aminotransferase (AST)	22 U/L	<40 U/L
Alkaline Phosphatase (ALP)	63 U/L	30–120 U/L
Total Bilirubin	0.5 mg/dL	0.2–1.2 mg/dL
C-Reactive Protein (CRP)	3 mg/L	<5 mg/L
Erythrocyte Sedimentation Rate (ESR)	11 mm/hr	0–20 mm/hr
Vitamin B12	360 pg/mL	180–914 pg/mL
Vitamin D	32 ng/mL	20–50 ng/mL
CSF Glucose	53 mg/dL	40–70 mg/dL
CSF Protein	28 mg/dL	15–45 mg/dL

**Figure 1:**
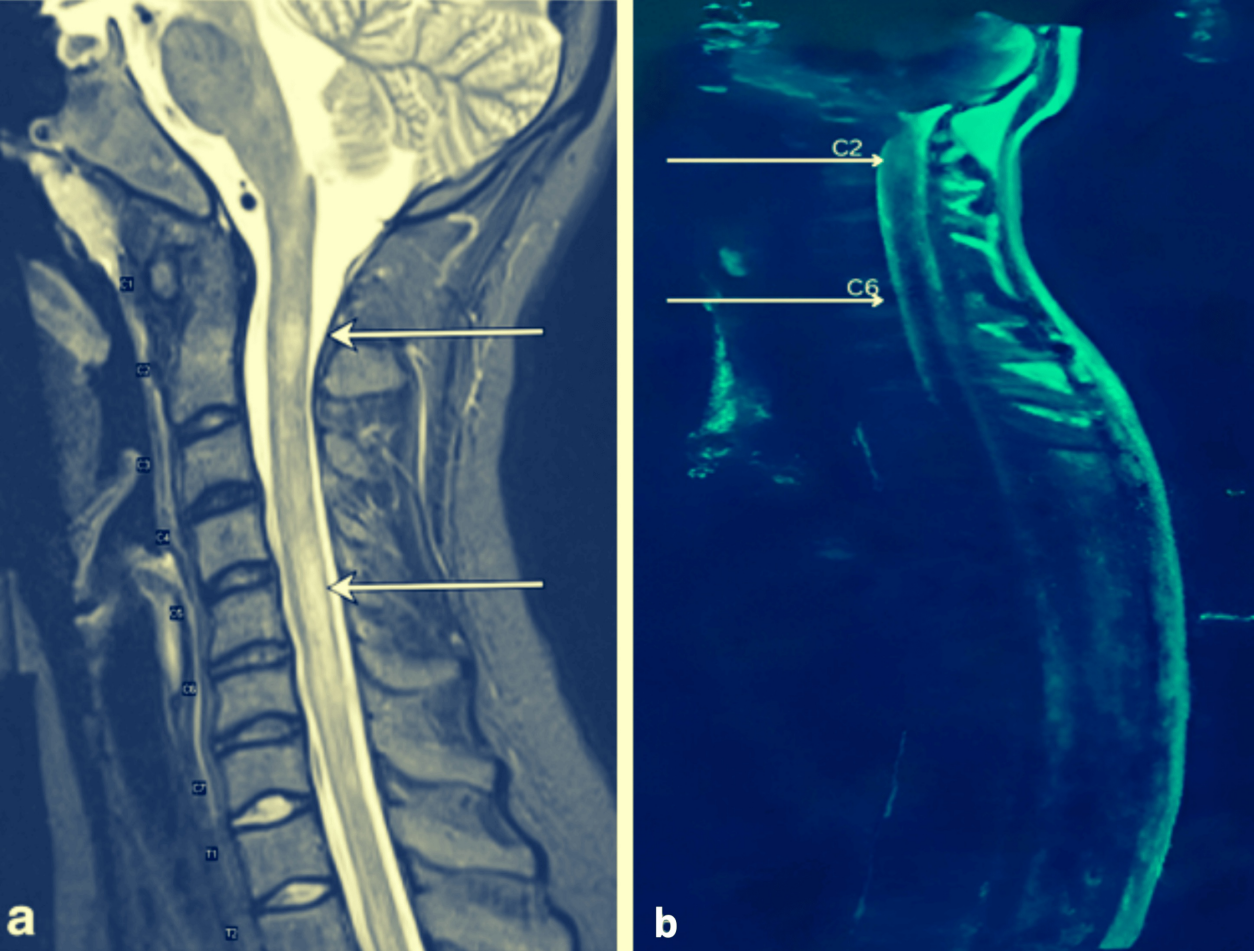
Cervical spine MRI in NMOSD: representative sagittal T2 (a) and our patient’s sagittal T2 (b). (a) Representative sagittal T2-weighted cervical MRI showing long-segment intramedullary T2 hyperintensity typical of neuromyelitis optica spectrum disorder (NMOSD). (b) Sagittal T2-weighted MRI from our patient, demonstrating C2-C6 intramedullary T2 hyperintensity with incomplete (central) transverse involvement (arrowheads), compatible with NMOSD partial transverse myelitis. Panel A is reproduced under CC BY 4.0 from [11]; minor edits (cropping) were made.

Cervical spine MRI (sagittal T2-weighted) showed an intramedullary hyperintensity from C2 to C6 with incomplete transverse involvement (incomplete transverse myelitis) (Figure 1). Brain CT was not performed. Brain MRI was reported as unremarkable; representative images were not retrievable from the archive for export at the time of submission. Serum AQP4-IgG was positive by a cell-based assay.

Acute treatment consisted of intravenous methylprednisolone 1,000 mg once daily for five days [12,13]; because early recovery was incomplete, intravenous immunoglobulin (IVIG) 0.4 g/kg/day for five days [12,13] was given. For relapse prevention, azathioprine was initiated with routine monitoring of CBC and LFTs [12]. Rituximab was planned as an escalation if relapse occurred or azathioprine was not tolerated; it was not given during the 24-week observation period. In our practice, pre-rituximab screening includes hepatitis B and C serology, HIV serology, tuberculosis screening, and baseline immunoglobulins and CD19/20 counts. These tests were not required during the reported period because rituximab was not administered.

The patient was reviewed at two, eight, and 24 weeks, with monitoring of visual acuity and color vision, optic disc assessment (with optical coherence tomography (OCT) when available), MRC strength, gait, bladder function, and relapse symptoms [13]. By week 24, she demonstrated clinical improvement, with enhanced lower-limb strength and improved bladder function, and no relapses were reported. The Expanded Disability Status Scale (EDSS) was not collected; functional recovery is therefore reported descriptively.

## Discussion

NMOSD now refers to a broad group of optic-spinal syndromes rather than only the classical combination of bilateral optic neuritis and longitudinally extensive transverse myelitis [5]. Consensus criteria published in 2023 emphasize that a single core attack (such as optic neuritis, myelitis, area-postrema syndrome, or brainstem syndrome) plus a positive AQP4 cell-based assay is sufficient to confirm the diagnosis, provided obvious “red-flag” patterns suggesting another disorder are absent [4]. Our patient’s unilateral optic neuritis and longitudinally extensive C2-C6 cervical cord lesion with incomplete transverse involvement fit these widened criteria, and similar atypical presentations now account for more than 15% of first attacks [8]. Longitudinally extensive transverse myelitis is typical of NMOSD, whereas the atypical feature in our case is unilateral optic neuritis at onset [4,5].

The epidemiology supports her profile, with a large systematic review showing the highest incidence and prevalence of NMOSD in African- and Asian-origin populations and a strong female bias [6,7]. This geographic pattern explains why clinicians working in the Gulf and North-East Africa must keep NMOSD in mind whenever they see an optic-spinal syndrome. Pathologically, AQP4-IgG binds astrocytes and activates complement, with secondary demyelination and axonal damage following close behind [3,8]. Even cervical lesions shorter than three vertebral segments can precipitate proximal corticospinal-tract degeneration, which clarifies why an incomplete lesion like ours can still produce marked leg weakness [14].

High-dose intravenous methylprednisolone is first-line for acute NMOSD attacks; if the steroid response is insufficient, early escalation to plasma exchange or intravenous immunoglobulin is recommended, particularly in AQP4-IgG-positive disease [13]. Rapid treatment is important. Observational data suggest that adding plasma exchange to steroids in severe relapses improves outcomes compared with steroids alone [15]. Where apheresis is not available, IVIG is an accepted escalation option [13] and has been reported to help in selected cases [16].

Long-term management differs sharply from multiple sclerosis: several MS disease-modifying treatments, including interferon-β and natalizumab, have worsened NMOSD or triggered severe attacks [10,17]. Instead, durable control relies on immunosuppression. Rituximab, mycophenolate mofetil, and azathioprine are traditional options, although their relative efficacy has never been directly compared [18,19]. Randomized trials of newer biologics (e.g., eculizumab and ravulizumab (complement inhibition) and satralizumab/tocilizumab (IL-6 receptor blockade)) demonstrate a reduction in relapse risk; however, access and cost remain limiting in many regions [13]. Rituximab, therefore, offered the best balance of effectiveness, price, and access for our patient.

The favorable eight-week recovery contrasts with historical cohorts in which more than half of untreated patients were blind or wheelchair-bound five years after onset [3]. Three elements made the difference: early suspicion of NMOSD despite an (atypical) MRI pattern, same-day AQP4 testing using a specific cell-based assay, and guideline-aligned escalation from steroids to IVIG followed by maintenance azathioprine. These steps are feasible even outside tertiary hospitals and can sharply cut the risk of permanent disability.

This case has two main limitations: it is a single-patient report with short follow-up (24 weeks), and we did not systematically collect standardized disability scores (EDSS) or quantitative OCT metrics.

## Conclusions

This case shows that even atypical presentations of NMOSD (such as unilateral optic neuritis with longitudinally extensive, incomplete transverse myelitis) require prompt diagnosis and timely therapy to prevent disability. In our AQP4-IgG-positive patient, acute steroids followed by IVIG and maintenance azathioprine were associated with clinical improvement and no relapses over 24 weeks in a resource-constrained setting. While LETM is typical of NMOSD, the unilateral optic neuritis at onset is atypical; recognizing this pattern can help avoid delays in care. A longer follow-up is needed to confirm the durability of remission.

## References

[1] Jarius S, Wildemann B (2013). The history of neuromyelitis optica. J Inflamm.

[2] Wingerchuk DM, Lennon V, Pittock S, Lucchinetti C, Weinshenker B (2006). Revised diagnostic criteria for neuromyelitis optica. Neurology.

[3] Wingerchuk DM, Lennon VA, Lucchinetti CF, Pittock SJ, Weinshenker BG (2007). The spectrum of neuromyelitis optica. Lancet Neurol.

[4] Jarius S, Aktas O, Ayzenberg I (2023). Update on the diagnosis and treatment of neuromyelits optica spectrum disorders (NMOSD)-revised recommendations of the Neuromyelitis Optica Study Group (NEMOS). Part I: diagnosis and differential diagnosis. Journal of neurology.

[5] Wingerchuk DM, Banwell B, Bennett JL (2015). International consensus diagnostic criteria for neuromyelitis optica spectrum disorders. Neurology.

[6] Papp V, Magyari M, Aktas O (2021). Worldwide incidence and prevalence of neuromyelitis optica: a systematic review. Neurology.

[7] Jarius S, Paul F, Weinshenker BG, Levy M, Kim HJ, Wildemann B (2020). Neuromyelitis optica. Nat Rev Dis Primers.

[8] Siriratnam P, Huda S, Butzkueven H, van der Walt A, Jokubaitis V, Monif M (2024). Risks and outcomes of pregnancy in neuromyelitis optica spectrum disorder: a comprehensive review. Autoimmun Rev.

[9] Li F, Sui X, Pan X (2024). Neuromyelitis optica spectrum disorder with ultra-longitudinally extensive transverse myelitis: a case report and literature review. Heliyon.

[10] Helmchen C, Buttler GM, Markewitz R, Hummel K, Wiendl H, Boppel T (2022). Acute bilateral optic/chiasm neuritis with longitudinal extensive transverse myelitis in longstanding stable multiple sclerosis following vector-based vaccination against the SARS-CoV-2. J Neurol.

[11] Mohajeri Moghaddam S, Bhatt AA (2018). Location, length, and enhancement: systematic approach to differentiating intramedullary spinal cord lesions. Insights Imaging.

[12] Chan KH, Lee CY (2021). Treatment of neuromyelitis optica spectrum disorders. Int J Mol Sci.

[13] Kümpfel T, Giglhuber K, Aktas O (2024). Update on the diagnosis and treatment of neuromyelitis optica spectrum disorders (NMOSD) - revised recommendations of the Neuromyelitis Optica Study Group (NEMOS). Part II: attack therapy and long-term management. J Neurol.

[14] Zhang C, Yao Y, Sun J (2025). Brain corticospinal tract abnormalities in aquaporin-4 seropositive neuromyelitis optica spectrum disorder with longitudinally extensive transverse myelitis. Brain Commun.

[15] Abboud H, Petrak A, Mealy M, Sasidharan S, Siddique L, Levy M (2016). Treatment of acute relapses in neuromyelitis optica: steroids alone versus steroids plus plasma exchange. Mult Scler J.

[16] Tavor Y, Herskovitz M, Ronen G, Balbir-Gurman A (2021). Longitudinally extensive transverse myelitis in a lupus-neuromyelitis optica overlap. Rambam Maimonides Med J.

[17] Papeix C, Vidal JS, de Seze J (2007). Immunosuppressive therapy is more effective than interferon in neuromyelitis optica. Mult Scler.

[18] Shimizu Y, Yokoyama K, Misu T (2007). Development of extensive brain lesions following interferon beta therapy in relapsing neuromyelitis optica and longitudinally extensive myelitis. J Neurol.

[19] Sherman E, Han MH (2015). Acute and chronic management of neuromyelitis optica spectrum disorder. Curr Treat Options Neurol.

